# Birth Weight, Head Circumference, and Prenatal Exposure to Acrylamide from Maternal Diet: The European Prospective Mother–Child Study (NewGeneris)

**DOI:** 10.1289/ehp.1205327

**Published:** 2012-10-23

**Authors:** Marie Pedersen, Hans von Stedingk, Maria Botsivali, Silvia Agramunt, Jan Alexander, Gunnar Brunborg, Leda Chatzi, Sarah Fleming, Eleni Fthenou, Berit Granum, Kristine B. Gutzkow, Laura J. Hardie, Lisbeth E. Knudsen, Soterios A. Kyrtopoulos, Michelle A. Mendez, Domenico F. Merlo, Jeanette K. Nielsen, Per Rydberg, Dan Segerbäck, Jordi Sunyer, John Wright, Margareta Törnqvist, Jos C. Kleinjans, Manolis Kogevinas, the NewGeneris Consortium

**Affiliations:** 1 Centre for Research in Environmental Epidemiology (CREAL), Barcelona, Spain; 2 IMIM (Hospital del Mar Research Institute), Barcelona, Spain; 3 CIBER Epidemiologia y Salud Pública (CIBERESP), Barcelona, Spain; 4 INSERM (National Institute of Health and Medical Research), Team of Environmental Epidemiology Applied to Reproduction and Respiratory Health, Institute Albert Bonniot, Grenoble, France; 5 Department of Materials and Environmental Chemistry, Environmental Chemistry Unit, Stockholm University, Stockholm, Sweden; 6 National Hellenic Research Foundation, Institute of Biological Research and Biotechnology, Athens, Greece; 7 Department of Food Safety and Nutrition, and; 8 Department of Chemical Toxicology, Division of Environmental Medicine, Norwegian Institute of Public Health, Oslo, Norway; 9 Department of Social Medicine, Faculty of Medicine, University of Crete, Heraklion, Greece; 10 Centre for Epidemiology and Biostatistics, Leeds Institute of Genetics, Health and Therapeutics, University of Leeds, Leeds, United Kingdom; 11 Department of Environmental Immunology, Norwegian Institute of Public Health, Oslo, Norway; 12 Section of Environmental Health, Department of Public Health, University of Copenhagen, Copenhagen, Denmark; 13 Epidemiology, Biostatistics, and Clinical Trials, National Cancer Research Institute, Genoa, Italy; 14 Department of Biosciences and Nutrition, Karolinska Institute, Novum, Huddinge, Sweden; 15 Department of Health and Experimental Sciences, University Pompeu Fabra, Barcelona, Spain; 16 Bradford Institute for Health Research, Bradford, United Kingdom; 17 Department of Toxicogenomics, Maastricht University, Maastricht, the Netherlands; 18 National School of Public Health, Athens, Greece

**Keywords:** biomarker, children, diet, intrauterine growth restriction, *in utero* exposure

## Abstract

Background: Acrylamide is a common dietary exposure that crosses the human placenta. It is classified as a probable human carcinogen, and developmental toxicity has been observed in rodents.

Objectives: We examined the associations between prenatal exposure to acrylamide and birth outcomes in a prospective European mother–child study.

Methods: Hemoglobin (Hb) adducts of acrylamide and its metabolite glycidamide were measured in cord blood (reflecting cumulated exposure in the last months of pregnancy) from 1,101 singleton pregnant women recruited in Denmark, England, Greece, Norway, and Spain during 2006–2010. Maternal diet was estimated through food-frequency questionnaires.

Results: Both acrylamide and glycidamide Hb adducts were associated with a statistically significant reduction in birth weight and head circumference. The estimated difference in birth weight for infants in the highest versus lowest quartile of acrylamide Hb adduct levels after adjusting for gestational age and country was –132 g (95% CI: –207, –56); the corresponding difference for head circumference was –0.33 cm (95% CI: –0.61, –0.06). Findings were similar in infants of nonsmokers, were consistent across countries, and remained after adjustment for factors associated with reduced birth weight. Maternal consumption of foods rich in acrylamide, such as fried potatoes, was associated with cord blood acrylamide adduct levels and with reduced birth weight.

Conclusions: Dietary exposure to acrylamide was associated with reduced birth weight and head circumference. Consumption of specific foods during pregnancy was associated with higher acrylamide exposure *in utero*. If confirmed, these findings suggest that dietary intake of acrylamide should be reduced among pregnant women.

Acrylamide is neurotoxic in humans and animals, and is classified as a probable human carcinogen [International Agency for Research on Cancer (IARC) 1994]. Occupational exposure and smoking were originally regarded as the main sources of exposure to acrylamide in humans (IARC 1994), but a decade ago it was unexpectedly discovered that acrylamide formed in a wide variety of carbohydrate-containing foods during frying or baking at high temperatures (Tareke et al. 2002). Worldwide concern about potential health effects of dietary exposure to acrylamide followed the finding of acrylamide in commonly consumed foods such as fried potatoes, potato chips, biscuits, breakfast cereals, and coffee [European Food Safety Authority (EFSA) 2010; Joint FAO/WHO (Food and Agriculture Organization/World Health Organization) Expert Committee on Food Additives (JECFA) 2011; Manson et al. 2005]. Prenatal exposure to acrylamide is of particular concern because reproductive and developmental toxicity of acrylamide has been reported in rodents, including dose-dependent body weight reduction and skeletal malformations in offspring exposed *in utero* (El Sayyad et al. 2011; Manson et al. 2005; Tyl and Friedman 2003).

Acrylamide and its metabolite glycidamide are reactive and may form adducts with nucleophilic sites in proteins and DNA. Glycidamide has a much higher reactivity toward DNA than acrylamide, and a considerably higher genotoxicity than the parent compound (Rice 2005). Hemoglobin (Hb) adducts can serve as biomarkers of internal dose in the blood for reactive compounds such as acrylamide (Törnqvist et al. 2002). Detection of Hb adducts from acrylamide in human umbilical cord blood (Schettgen et al. 2004; von Stedingk et al. 2011), and studies of acrylamide in *ex vivo* placenta perfusion studies (e.g., Annola et al. 2008), show that acrylamide crosses the human placenta.

In view of the possible health effects associated with the widespread dietary exposure to acrylamide (EFSA 2010; IARC 1994; JECFA 2011; Manson et al. 2005) starting *in utero* (Brantsaeter et al. 2008), we investigated the association between prenatal exposure to acrylamide, measured as Hb adducts in cord blood, and birth outcomes in a multicenter European study. We also assessed prenatal exposure to acrylamide through maternal food frequency questionnaires (FFQs) and associations between maternal dietary exposure to acrylamide, cord blood adduct levels, and birth outcomes, hypothesizing that higher maternal intake of acrylamide-rich food during pregnancy would be associated with reduced intrauterine growth.

## Methods

*Study population.* The study was conducted by the NewGeneris consortium (www.newgeneris.org) as part of research exploring the impact of diet during pregnancy on child health (Merlo et al. 2009). Pregnant women enrolled during 2006–2010 in 11 maternity units located in Copenhagen, Denmark; Heraklion, Greece; Oslo and Akershus, Norway; Barcelona and Sabadell, Spain; and Bradford, England (Chatzi et al. 2012; Magnus et al. 2006; Pedersen et al. 2009; Raynor et al. 2008). Specific eligibility criteria were applied in the baseline cohorts for the participation of mothers [for details, see Supplemental Material, Table S1 (http://dx.doi.org/10.1289/ehp.1205327)]. Precise participation rates for the present analysis cannot be estimated because several filters were applied for inclusion of mothers in the NewGeneris study, such as giving birth during the periods of cord blood collection and processing, getting sufficient volume of cord blood, successful blood processing, and analyzing biomarkers.

Questionnaire information on maternal characteristics and cord blood Hb adduct measurements was available for 1,151 mother–child pairs. We excluded 16 pairs of twins and 34 mother–child pairs with missing information on maternal smoking, gestational age, birth weight, and/or sex of the child, leaving 1,101 mother–child pairs for analysis. Information on birth weight, head circumference, gestational age, sex, and mode of delivery was obtained from maternity records.

Gestational age for participants from Denmark, Greece, and Spain was estimated from the interval between last menstrual period and date of the delivery and was corrected by ultrasound measurement if there was a discordance of ≥ 7 days between both estimates. Ultrasound-based estimation was provided for most participants from England and Norway. We defined small-for-gestational-age (SGA) children as those who weighed less than the 10th percentile of the cohort-specific reference of fetal growth (mean birth weight for all SGA children: 2,838 g), stratified by completed week of gestation and sex.

Ethical approval was obtained from the research ethics committee in each country: the Regional Ethical Review Board in Stockholm, Sweden; the Capital Region of Denmark; the Ethical Committee of the University Hospital in Heraklion, Crete, Greece; the Norwegian Regional Committee for Medical and Health Research Ethics; the Clinical Research Ethics Committee of Barcelona, Spain; and the Bradford Local Research Ethics Committee, Bradford, United Kingdom. Further details regarding ethics approval are provided in Supplemental Material (http://dx.doi.org/10.1289/ehp.1205327). Written informed consent for participation of the women and their children was obtained from all participating women.

*Acrylamide and glycidamide Hb adducts in cord and maternal blood.* Cord blood was collected in heparin tubes from the placenta by umbilical puncture immediately after delivery, following a common protocol at each site. Erythrocytes were separated by centrifugation and stored at –20°C before and after shipment on dry ice. Hb adducts from acrylamide and glycidamide were simultaneously determined by the “adduct FIRE procedure” (fluorescein isothiocyanate R Edman) in 1,101 cord blood samples and 172 maternal blood samples (von Stedingk et al. 2010, 2011). In brief, adducted N-terminal valines were detached using the Edman reagent fluoresceine-5-isothiocyanate. The detached analytes were purified by solid phase extraction and analyzed by liquid chromatography/mass spectrometry.

Ethylene oxide Hb adduct levels that are associated with exposure to tobacco smoke were also measured in 1,074 cord blood samples (to assess exposure to tobacco smoke during pregnancy) using the same methods (von Stedingk et al. 2011).

*Maternal diet.* Detailed information on maternal diet during pregnancy was obtained from the mothers using FFQs collected before or at the time of delivery (Merlo et al. 2009). We grouped food and drink items into eight groups known to contain potentially high levels of acrylamide based on similarities in the composition and processing of the foods in each group—specifically, fried potatoes, potato chips, breakfast cereals, crisp bread, coffee, cookies, fine bakery products, bread and toast (Brantsaeter et al. 2008; EFSA 2010; JECFA 2011). We developed an acrylamide food score for nonsmokers following the same approach used for the evaluation of the Mediterranean diet (Trichopoulou et al. 2003). The score was based on consumption of the eight food groups (as mentioned above); each nonsmoking woman received a score of 0 for each food if her consumption was below the country-specific median, and a score of 1 if her consumption was above the median. Scores were then added for each woman and ranged from 0 (lowest intake) to 8 (highest). In all dietary analyses we excluded women with missing FFQs (*n* = 146), those who smoked during the last 4 months of pregnancy (*n* = 129), and 25 women with a total energy estimate of < 500 or > 6,000 kcal/day (Butte and King 2005; Willett 1998).

*Statistical analysis.* We used linear regression models for birth outcomes evaluated on a continuous scale (birth weight, head circumference) and calculated beta-coefficients and 95% confidence intervals (CIs). Logarithmic-binomial regression models were used to determine relative risks (RRs) and 95% CIs for SGA. Cord blood Hb adduct levels were modeled on a continuous scale (effect estimated per 10 pmol/g Hb increments) or categorized according to quartiles of the distribution. All models were adjusted for country of the child’s birth and gestational age (continuous in completed weeks). Additional adjustment for gestational age as a quadratic term (continuous completed weeks squared) gave nearly identical results (not shown). Further adjustment was based on *a priori* selection of potential risk factors for reduced birth weight including maternal smoking (no, yes), passive smoking (no, yes), sex, prepregnancy body mass index (BMI; kilograms per meter squared), parity (0, ≥ 1), maternal age (years), maternal ethnicity (white, nonwhite), and maternal education (low, middle, high) as a marker of socioeconomic position. In addition, models were adjusted for high or low maternal consumption during pregnancy (according to country-specific median intake) of fruit and vegetables (based on 20–61 questionnaire items), fish (fresh, canned, dried, and prepared fish and shellfish, including items with high and low fat content), and soft drinks (e.g., cola, other soft drinks, energy soft drinks including regular and light products) as markers of more versus less healthy dietary patterns.

The average lifespan of erythrocytes is 4 months in adults (Törnqvist et al. 2002). Therefore, women who never smoked or who quit smoking before the last 4 months of pregnancy were categorized as “nonsmokers,” and those who never smoked were categorized as “never smokers.” All associations were examined for the full study population (*n* = 1,101) and separately for children born to nonsmokers (*n* = 972).

In addition to estimating associations adjusted for country, we estimated country-specific associations between acrylamide Hb adducts and birth weight, and performed a meta-analysis to derive pooled estimates of effect. Only results from fixed-effects models are shown because heterogeneity of effects between centers were not statistically significant.

Associations of maternal diet with acrylamide Hb adduct levels in cord blood, and with birth weight, were estimated using linear regression models that were adjusted as described above. Exposure–response curves were estimated using a generalized additive model with the dietary score modeled as a smoothed spline with 2 degrees of freedom, adjusted for country and gestational age. We used alpha levels of 5% as reference value for statistical significance and Stata S.E. version 10.0 for the statistical analyses (StataCorp, College Station, TX, USA).

## Results

*Levels of Hb adducts from acrylamide.* Acrylamide and glycidamide Hb adducts in cord blood were detectable in all children (*n* = 1,101). The median Hb adduct level from acrylamide was 14.4 pmol/g Hb (range, 4.4–147.6) and for glycidamide was 10.8 pmol/g Hb (range, 2.0–117.6). There was a statistically significant correlation between glycidamide and acrylamide adduct levels in cord blood (Pearson correlation coefficient *r* = 0.85, *p* < 0.001). Median acrylamide adduct levels were higher in cord blood from children of mothers who smoked (*n* = 129) than in children of nonsmokers (*n* = 972; 30.5 vs. 13.8 pmol/g Hb, *p* < 0.001). Corresponding levels for glycidamide adducts were 20.7 versus 10.1 pmol/g Hb (*p* < 0.001), and for ethylene oxide adducts (as a marker of exposure to cigarette smoke) were 24.5 versus 8.9 pmol/g Hb, (*p* < 0.001).

**Figure 1:**
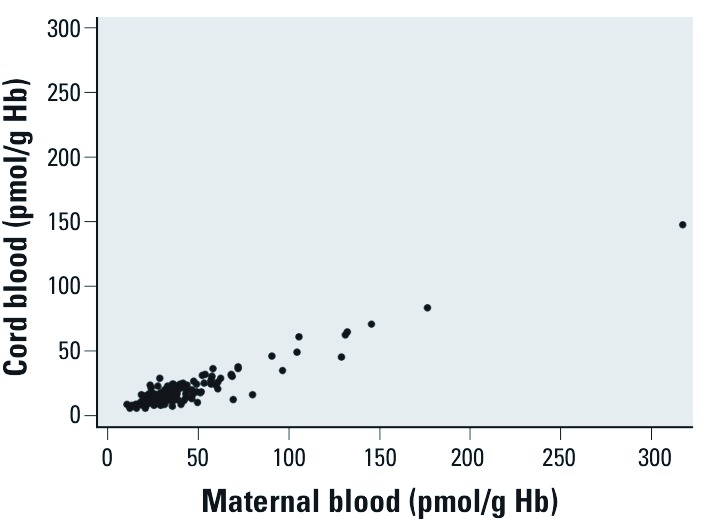
Acrylamide Hb adduct levels (pmol/g Hb) in mother–child pairs (*n* = 172).

The median acrylamide Hb adduct levels in cord blood were approximately half of the levels in paired maternal blood (Figure 1). Hb adduct levels in cord blood were positively correlated with both maternal acrylamide (*r* = 0.95, *p* < 0.001, *n* = 171) and glycidamide Hb adducts (*r* = 0.94, *p* < 0.001, *n* = 171). Among nonsmokers the highest median level of acrylamide adducts was detected in children from England (23.6 pmol/g Hb) and the lowest in children from Denmark (12.0 pmol/g Hb).

*Factors associated with birth weight.* The participating mothers were mainly white, nonsmoking, in their late 20s to early 30s with prepregnancy BMI in a range of 18.5–24.9 kg/m^2^ and already parous (Table 1). The prevalence of smokers was lowest in Norway and Denmark (2.4% and 3.8%, respectively) and highest in Greece (21%). Birth weight was significantly increased in association with maternal age, parity (parous vs. nulliparous), prepregnancy BMI, nonsmoking status, male sex, and gestational age (Table 2). On average, children from the Northern European countries weighed more than children from Spain and Greece (*p* < 0.001).

**Table 1:** Study population characteristics (*n* = 1,101) [*n* (%) or mean ± SD].

Characteristics	All (n = 1,101)	Nonsmokersa (n = 972)
Country		
Greece	236 (21)	186 (19)
Spain	220 (20)	185 (19)
Norway	247 (22)	241 (25)
England	186 (17)	156 (16)
Denmark	212 (19)	204 (21)
Maternal ethnicity		
White	912 (83)	792 (82)
Nonwhite	187 (17)	179 (18)
Missing (n)	2	1
Maternal age	30.9 ± 5.2	31.1 ± 5.1
Maternal education		
High	357 (36)	342 (39)
Middle	371 (38)	329 (38)
Low	251 (26)	207 (24)
Missing (n)	122	94
Parity		
Nulliparous	388 (36)	356 (37)
Parous	688 (64)	594 (63)
Missing (n)	25	22
Prepregnancy BMI (kg/m2)	24.1 ± 4.9	24.0 ± 4.8
Missing (n)	110	89
Secondhand smoke during pregnancy		
No	652 (65)	622 (70)
Yes	354 (35)	272 (30)
Missing (n)	95	78
Sex
Boys	550 (50)	489 (50)
Girls	551 (50)	483 (50)
Gestational age (completed weeks)		
< 37	38 (3)	33 (3)
≥ 37	1,063 (97)	939 (97)
Birth weight (g)		
< 2,500	18 (2)	11 (1)
≥ 2,500	1,083 (98)	961 (99)
SGA		
No	819 (92)	734 (92)
Yes	72 (8)	60 (8)
Missing (n)	210	178
Birth head circumference (cm)	34.8 ± 1.5	34.8 ± 1.5
Missing (n)	96	82
Cord blood hemoglobin adduct (pmol/g Hb)		
Acrylamide	19.7 ± 16.5	16.8 ± 11.1
Glycidamide	13.6 ± 10.1	11.8 ± 6.6
Ethylene oxide	13.2 ± 13.6	10.5 ± 6.7
Missing (n)	27	24
aWomen who never smoked or who quit smoking before the last 4 months of pregnancy.		

**Table 2:** Differences in birth weight according to sociodemographic, reproductive, and lifestyle factors.

Characteristic	Total population (n = 1,101)					Nonsmokersa (n = 972)				
	n	β (95%CI)	p-Value	n	β (95%CI)	p-Value
Country
Greece	236	Ref: 3,205 g	186	Ref: 3,219 g
Spain	220	78 (–1, 157)	0.050	185	81 (–4, 185)	0.063
Norway	247	197 (117, 277)	< 0.001	241	203 (119, 286)	< 0.001
England	186	258 (178, 337)	< 0.001	156	228 (140, 316)	< 0.001
Denmark	212	256 (178, 337)	< 0.001	204	258 (175, 341)	< 0.001
Ethnicity (white vs. nonwhite)	1,099	11 (–69, 91)	0.79	972	–39 (–123, 46)	0.37
Maternal age (years)	1,101	6 (1, 11)	0.015	972	5 (0, 10)	0.086
Maternal education
High	357	Ref: 3,512 g	342	Ref: 3,523 g
Middle	371	–54 (–114, 7)	0.082	329	–58 (–122, 6)	0.074
Low	251	–51 (–120, 17)	0.143	207	–39 (–111, 34)	0.30
Parity (nulliparous vs. parous)	1,076	115 (61, 170)	< 0.001	950	110 (54, 166)	< 0.001
Prepregnancy BMI (kg/m2)	991	12 (7, 18)	< 0.001	883	12 (6, 18)	< 0.001
Maternal smoking (no vs. yes)	1,101	–142 (–221, –63)	< 0.001
Passive smoke (no vs. yes)	1,006	–39 (–101, 23)	0.22	894	7 (–60, 73)	0.85
Ethylene oxide (10 pmol/g Hb)	1,074	–31 (–50, –12)	0.001	948	–27 (–67, 13)	0.185
Sex (boy vs. girl)	1,101	–140 (–189, –91)	< 0.001	972	–141(–193, –90)	< 0.001
Gestational age (weeks)	1,101	113 (93, 192)	< 0.001	972	110 (91, 130)	< 0.001
Ref, reference. Beta-coefficients (β) correspond to the difference in birth weight in grams and are estimated from linear regression models adjusted for country and gestational age (completed weeks). aWomen who never smoked or who quit smoking before the last 4 months of pregnancy.												

*Hb adduct levels and birth weight.* Higher levels of acrylamide and glycidamide adducts in cord blood were associated with a significant decrease in birth weight (Table 3). The mean birth weight was reduced by 35 g (95% CI: –51, –19 g) with each 10-pmol/g Hb increase in acrylamide adduct levels after adjusting for country of birth and gestational age in the total population, and by 20 g (95% CI: –46, 6 g) among 972 nonsmokers. A 10-pmol/g Hb increase in glycidamide adducts was associated with a 60-g reduction in birth weight (95% CI: –87, –34 g) in the total population, and a 53-g reduction (95% CI: –95, –10 g) in nonsmokers. Results were similar among the subgroup of 889 women who were never-smokers, with a mean birth weight reduction of 26 g (95% CI: –54, 3 g; *p* = 0.074) for a 10-pmol/g Hb increase in acrylamide adducts when adjusted for gestational age and country of birth; the corresponding reduction in birth weight for glycidamide adducts was 65 g (95% CI: –111, –19 g).

**Table 3:** Prenatal exposure to acrylamide and glycidamide measured as Hb adducts in cord blood, and associations with birth weight.

Variable	Acrylamide Hb adducts					Glycidamide Hb adducts				
	n	β (95%CI)	p-Value	n	β (95%CI)	p-Value
Basic adjusteda
All
Change per 10 pmol/g Hb	1,101	–35 (–51, –19)	< 0.001	1,100	–60 (–87, –34)	< 0.001
Quartile 1 (lowest)	288	Ref: 3,460 g	283	Ref: 3,492 g
Quartile 2	263	–65 (–136, 5)	0.066	269	–53 (–124, 18)	0.145
Quartile 3	275	–110 (–180, –39)	0.002	276	–61 (–131, 11)	0.094
Quartile 4 (highest)	275	–132 (–207, –56)	0.001	272	–136 (–212, –60)	0.001
Nonsmokers
Change per 10 pmol/g Hb	972	–20 (–46, 6)	0.187	972	–53 (–95, –10)	0.016
Quartile 1 (lowest)	247	Ref: 3,445 g	249	Ref: 3,503 g
Quartile 2	242	–26 (–99, 48)	0.50	239	–73 (–147, 1)	0.053
Quartile 3	241	–105 (–181, –31)	0.006	244	–76 (–150, –1)	0.046
Quartile 4 (highest)	242	–107 (–188, –27)	0.009	239	–103 (–182, –23)	0.012
Further adjustedb
All
Change per 10 pmol/g Hb	747	–23 (–51, 5)	0.10	746	–22 (–67, 23)	0.33
Quartile 1 (lowest)	208	Ref: 3,509 g	214	Ref: 3,527 g
Quartile 2	194	–65 (–139, 19)	0.14	199	–80 (–159, –1)	0.046
Quartile 3	205	–110 (–207, –48)	0.002	189	–50 (–131, –31)	0.022
Quartile 4 (highest)	140	–157 (–256, –58)	0.002	144	–110 (–207, –12)	0.028
Nonsmokers
Change per 10 pmol/g Hb	675	–34 (–72, 4)	0.078	674	–52 (–112, 8)	0.088
Quartile 1 (lowest)	174	Ref: 3,445 g	186	Ref: 3,542 g
Quartile 2	183	–19 (–102, 64)	0.65	180	–67 (–150, 16)	0.12
Quartile 3	190	–132 (–216, –49)	0.002	171	–89 (–173, –4)	0.035
Quartile 4 (highest)	128	–149 (–248, –50)	0.003	137	–97 (–193, –1)	0.05
Ref, reference. Beta coefficients (β) are from linear regression analyses and correspond to change in birth weight (grams) per 10 pmol/g Hb adducts, or relative to the lowest quartile of acrylamide or glycidamide adduct levels. Acrylamide adduct quartiles for all: ≤ 10.9, > 10.9 – ≤ 14.4, > 14.4 – ≤ 21.7, > 21.7; for nonsmokers: ≤ 10.5, > 10.5 – ≤ 13.8, > 13.8 – ≤ 19.2, > 19.2 pmol/g Hb. Glycidamide adduct quartiles for all: ≤ 7.9, > 7.9 – ≤ 10.8, > 10.8 – ≤ 15.7, > 15.7; for nonsmokers: ≤ 7.6, > 7.6 – ≤ 10.1, > 10.1 – ≤ 14.2, >14.2 pmol/g Hb. aAdjusted for gestational age (completed weeks) and country. bAdditionally adjusted for maternal smoking at the end of pregnancy (no, yes), passive smoking (no, yes), sex (boy, girl), prepregnancy BMI (kg/m2), parity (0, ≥ 1), maternal age (years), maternal ethnicity (white, nonwhite), maternal education (low, middle, high), and maternal consumption of fruit and vegetables, fish, and soft drinks (low, high).												

Birth weight decreased monotonically with increasing quartiles of exposure (Table 3). The estimated difference in birth weight for infants in the highest versus lowest quartile of acrylamide Hb adduct levels after adjusting for gestational age and country was –132 g (95% CI: –207, –56 g) in the total population and –107 g (95% CI: –188, –27 g) when restricted to nonsmokers. The estimated difference in birth weight for infants in the highest versus lowest quartile of glycidamide adducts was –136 g (95% CI: –212, –60 g) in the total population and –103 g (95% CI: –182, –23 g) among nonsmokers (Table 3). Among term deliveries (*n* = 1,063), the difference in birth weight for children in the highest versus lowest quartile of acrylamide Hb adduct levels was –137 g (95% CI: –214, –60 g); a similar difference was observed for 939 term babies of nonsmokers (–120 g; 95% CI: –201, –38 g).

Associations between Hb adducts and birth weight adjusted for country and gestational age were not substantially modified when further adjustments were made for other potential risk factors for reduced birth weight, including sex, prepregnancy BMI, parity, maternal age, ethnicity, education, passive smoking and smoking, and dietary variables that could be related with a healthy (or unhealthy) eating pattern such as intakes of vegetables and fruits, fish, or soft drinks (Table 3). The population in the comprehensively adjusted models (*n* = 747) is smaller than the population in models adjusted only for country and gestational age (*n* = 1,101). We also estimated associations adjusted for ethylene oxide Hb adduct levels in cord blood as a biomarker for active/passive smoking, in addition to the other covariates listed above. The difference in birth weight for infants of nonsmokers (*n* = 656) in the highest versus lowest quartile of acrylamide Hb adduct levels remained statistically significant after adjustment for ethylene oxide adducts (–142 g; 95% CI: –246, –38 g), whereas the corresponding difference was borderline significant for glycidamide Hb adducts (–82 g; 95% CI: –182, 17 g; *p* = 0.11).

**Figure 2:**
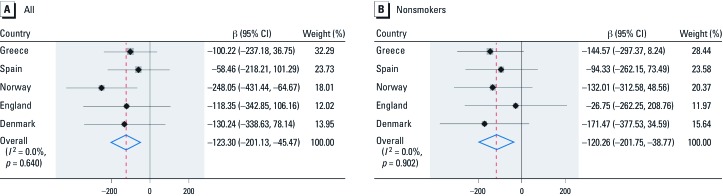
Forest plot of the association between acrylamide hemoglobin adducts (highest relative to the lowest quartile*^a^*) and birth weight by country and combined meta-analytic estimate, adjusted for gestational age (completed weeks) in the full population (*A*), and in nonsmokers (*B*). Gray shaded areas superimposed over the country-specific point estimates are proportional to the country-specific weights used in the meta-analyses, and the associated 95% CIs are shown as horizontal black lines. The summary β, which corresponds to the change in birth weight (grams) for the highest relative to the lowest quartile of acrylamide, is indicated with a red dashed vertical line and blue diamond, and the associated 95% CIs are indicated by the lateral tips of the diamond. The solid vertical line refers to no change in birth weight. The names of the countries are shown on the left and the country-specific βs, 95% CIs, and weights of each study on the right. Full population (*n* = 1,101): test for heterogeneity, Q = 2.5 on 4 degrees of freedom (*p* = 0.640). Nonsmokers (*n* = 972): test for heterogeneity, Q = 1.0 on 4 degrees of freedom (*p* = 0.902). *^a^*Acrylamide adduct quartiles for all: ≤ 10.9 vs. > 21.7 and for nonsmokers: ≤ 10.5 vs. > 19.2 pmol/g Hb.

Negative associations between acrylamide and glycidamide Hb adduct levels in cord blood and birth weight were observed for all countries (Figure 2). A meta-analysis of country-specific estimates gave similar results to estimates based on combined data adjusted for country, with a 10-pmol/g Hb increase in acrylamide adducts associated with a 36-g (95% CI: –53, –19 g) decrease in birth weight, and a 10-pmol/g Hb increase in glycidamide adduct levels associated with a 63-g (95% CI: –90, –36 g) decrease in birth weight.

The pattern was also consistent among nonsmokers (–26 g, 95% CI: –52, 1 g, and –63 g, 95% CI: –106, –20 g for a 10-pmol/g Hb increase in acrylamide and glycidamide, respectively), with no significant heterogeneity between countries (*p* = 0.51 and *p* = 0.46), although in England the association with birth weight was minimal (–2 g, 95% CI: –44, 41 g, and –18 g, 95% CI: –95, 59 g).

*Hb adduct levels and SGA.* SGA (birth weight < 10th percentile for the cohort according to week of gestation and sex) was increased in association with a 10-pmol/g Hb increase in acrylamide adduct levels in the full population (RR = 1.20; 95% CI: 1.08, 1.33, based on 891 observations and 72 SGA births) and for infants of nonsmokers (RR = 1.35; 95% CI: 1.10, 1.65, based on 794 observations and 60 SGA births). The corresponding estimates for glycidamide were RR = 1.36 (95% CI: 1.13,1.64) for all and RR = 1.42 (95% CI: 1.00, 2.02) for nonsmokers.

*Hb adduct levels and birth head circumference.* The highest versus lowest quartile of acrylamide Hb adduct levels was associated with a significant reduction in head circumference of 0.33 cm (95% CI: –0.61, –0.06 cm) in the full population and among nonsmokers, with similar results observed for glycidamide (Table 4). Similar to the associations with birth weight, there was a monotonic reduction in birth head circumference with increasing quartiles of exposure. This pattern was also found after further adjustment in the smaller study population with available information on potential risk factors (Table 4). These associations, however, were not statistically significant when adjusted for the other potential risk factors, as above.

**Table 4:** Prenatal exposure to acrylamide and glycidamide measured as hemoglobin (Hb) adducts in cord blood, and associations with birth head circumference.

Variable	Acrylamide Hb adducts					Glycidamide Hb adducts				
	n	β (95%CI)	p-Value	n	β (95%CI)	p-Value
Basic adjusteda
All
Change per 10 pmol/g Hb	1,005	–0.06 (–0.12, 0.00)	0.034	1,004	–0.10 (–0.20, 0.00)	0.040
Quartile 1 (lowest)	272	Ref: 34.89 cm	268	Ref: 34.99 cm
Quartile 2	237	–0.10 (–0.35, 0.15)	0.44	251	–0.03 (–0.29, 0.22)	0.79
Quartile 3	251	–0.18 (–0.43, 0.08)	0.17	251	–0.14 (–0.40, 0.11)	0.27
Quartile 4 (highest)	245	–0.33 (–0.61, –0.06)	0.018	234	–0.38 (–0.65,–0.10)	0.007
Nonsmokers
Change per 10 pmol/g Hb	890	–0.07 (–0.16, 0.03)	0.184	889	–0.16 (–0.33, 0.00)	0.049
Quartile 1 (lowest)	232	Ref: 34.86 cm	235	Ref: 35.05 cm
Quartile 2	220	–0.02 (–0.29, 0.25)	0.88	222	–0.15 (–0.42, 0.12)	0.27
Quartile 3	222	–0.13 (–0.40, 0.14)	0.36	224	–0.20 (–0.47, 0.07)	0.14
Quartile 4 (highest)	216	–0.35 (–0.65, –0.05)	0.021	208	–0.34 (–0.63, –0.05)	0.023
Further adjustedb
All
Change per 10 pmol/g Hb	713	0.02 (–0.08, 0.12)	0.71	712	–0.01 (–0.17, 0.16)	0.93
Quartile 1 (lowest)	201	Ref: 34.98 cm	208	Ref: 35.08 cm
Quartile 2	182	–0.08 (–0.37, 0.21)	0.57	192	–0.08 (–0.36, 0.21)	0.60
Quartile 3	198	–0.08 (–0.37, 0.21)	0.60	177	–0.07 (–0.36, 0.23)	0.66
Quartile 4 (highest)	132	–0.22 (–0.59, 0.14)	0.23	135	–0.26 (–0.62, 0.09)	0.15
Nonsmokers
Change per 10 pmol/g Hb	645	–0.05 (–0.09, 0.19)	0.51	644	–0.05 (–0.27, 0.17)	0.64
Quartile 1 (lowest)	168	Ref: 34.95 cm	181	Ref: 35.14 cm
Quartile 2	173	0.01 (–0.30, 0.32)	0.96	173	–0.08 (–0.38, 0.22)	0.61
Quartile 3	180	–0.10 (–0.41, 0.21)	0.52	160	–0.21 (–0.52, 0.10)	0.19
Quartile 4 (highest)	124	–0.21 (–0.57, 0.16)	0.27	130	–0.23 (–0.58, 0.12)	0.20
Ref, reference. Beta coefficients (β) are from linear regression analyses and correspond to change in birth head circumference (cm) per 10 pmol/g Hb adducts, or relative to the lowest quartile of acrylamide or glycidamide adduct levels. Acrylamide adduct quartiles for all: ≤ 10.9, > 10.9 – ≤ 14.4, > 14.4 – ≤ 21.7, > 21.7; for nonsmokers: ≤ 10.5, > 10.5 – ≤ 13.8, > 13.8 – ≤ 19.2, > 19.2 pmol/g Hb. Glycidamide adduct quartiles for all: ≤ 7.9, > 7.9 – ≤ 10.8, > 10.8 – ≤ 15.7, > 15.7; for nonsmokers: ≤ 7.6, > 7.6 – ≤ 10.1, > 10.1 – ≤ 14.2, > 14.2 pmol/g Hb. aAdjusted for gestational age (completed weeks) and country. bAdditionally adjusted for maternal smoking at the end of pregnancy (no, yes), passive smoking (no, yes), sex (boy, girl), prepregnancy BMI (kg/m2), parity (0, ≥ 1), maternal age (years), maternal ethnicity (white, nonwhite), maternal education (low, middle, high) and maternal consumption of fruit and vegetables, fish, and soft drinks (low, high).												

**Figure 3:**
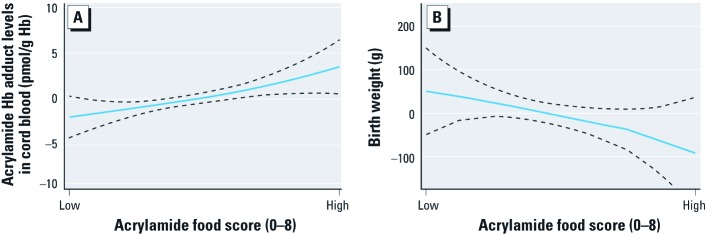
Association of maternal acrylamide exposure through diet among nonsmokers (*n* = 801) estimated through an acrylamide-rich food score with acrylamide hemoglobin adducts in cord blood (*A*) and birth weight (*B*). Generalized additive model with a smoothed spline for acrylamide-rich food score adjusting for country, and gestational age (completed weeks). Dashed lines are 95% CIs.

*Maternal diet, acrylamide Hb adduct levels in cord blood, and birth weight.* A 1-unit increase in the acrylamide-rich food score was associated with higher Hb cord blood adduct levels (Figure 3) for acrylamide (0.68 pmol/g Hb; 95% CI: 0.30, 1.06) and glycidamide (0.39 pmol/g Hb; 95% CI: 0.15, 0.63) based on food score modeled as a simple continuous variable. Consistent with the associations observed between Hb adducts from acrylamide and birth weight, a 1-unit increase in the acrylamide food score was associated with a 16-g decrease in birth weight (95% CI: –33, 1; *p* = 0.066) after adjustment for country and gestational age. Additional adjustment did not change this association (Figure 3). Higher food scores were associated with a nonsignificant reduction in birth head circumference of –0.01 cm (95% CI: –0.07, 0.05 cm; *p* = 0.72) among children of nonsmoking women with acrylamide food score data (*n* = 726).

## Discussion

This study provides strong evidence that higher prenatal exposure to acrylamide through maternal diet during pregnancy is associated with reduced birth weight and head circumference. Prenatal exposure to acrylamide was evaluated using Hb adducts, which are well-established biomarkers for acrylamide exposure (Vikström et al. 2011). Birth weight decreased monotonically with increasing acrylamide and glycidamide adduct cord blood levels. These findings were consistent between countries and were shown in the full study population as well as in nonsmokers. Maternal consumption of acrylamide-rich foods was associated with higher levels of acrylamide and glycidamide Hb adducts in cord blood and with lower birth weight.

Prenatal exposure assessment using biomarkers is a key strength of the study. The measurement of Hb adduct levels in cord blood enabled a more accurate estimation of prenatal exposure to acrylamide compared with estimates based solely on dietary questionnaires or maternal Hb adduct levels. When cord blood Hb adduct levels are measured, variation related to transplacental exposure, uptake, and metabolism among children is taken into account. In line with our observation of higher Hb adduct levels in children from England compared with those of other study countries, higher levels have also been reported in nonsmoking British adults compared with other European adults (Vesper et al. 2008). Furthermore, our study population was large, and detailed information on maternal characteristics, including smoking and dietary habits during pregnancy, was collected in a manner that enabled us to evaluate potential sources of exposure contributing to *in utero* formation of Hb adducts and reduce potential biases through adjustment in a large subset of the study population.

In addition to dietary intake of acrylamide (Brantsaeter et al. 2008; Tran et al. 2010; Vikström et al. 2011), it is possible that acrylamide adducts were acting as a proxy marker for another dietary exposure or mix of exposures that were responsible for the associations observed, such as other Maillard reaction products that, like acrylamide, are formed during processing of food at high temperatures (Chaundhry et al. 2006; Tareke et al. 2002). Alternatively, acrylamide adducts may have been acting as a marker of a less healthy diet in general. Acrylamide may also be one of many contributors to the observed association. Adjusting for indicators of healthy and unhealthy eating habits (such as fruits and vegetables, fish, and soft drinks), maternal BMI, and indicators of socioeconomic status, did not substantially alter associations among the subset of the population with available data. Furthermore, foods that are generally considered to be part of a healthy diet, such as crisp bread and certain breakfast cereals, may contain high concentrations of acrylamide (EFSA 2010). Finally, Hb acrylamide and glycidamide adducts could also reflect exposure to tobacco smoke (von Stedingk et al. 2011). Monotonic dose–response associations of adduct levels with birth outcomes were observed in women who were nonsmokers in pregnancy, as well as in never-smokers, even after adjusting for passive smoking based on self-reporting or using ethylene oxide adducts as biomarkers of exposure to tobacco smoke. We cannot rule out uncontrolled confounding or the possibility that adduct levels are serving as a proxy marker for some other causal factor; however, given the consistency of our findings in different population subgroups and after adjustment for multiple potential confounders, it seems unlikely that prenatal exposure to tobacco smoke or other dietary compounds could fully explain the associations observed between acrylamide and birth weight.

A limitation of exposure assessment based on dietary information is the difficulty of evaluating exposure to toxic agents, such as acrylamide, for which concentrations may vary substantially among similar foods, depending on manufacturing or preparation methods (EFSA 2010; JECFA 2011; Manson et al. 2005; Tareke et al. 2002). Quantitative estimation of dietary intake is particularly complex in international studies. Nonetheless, intakes of key food types have been found to predict higher acrylamide Hb adduct levels in other study populations (Tran et al. 2010; Vikström et al. 2011) as well as in the present study population.

Decreases in offspring body weight following maternal acrylamide exposure during gestation have been consistently observed in mice and rats (El Sayyad et al. 2011; Tyl and Friedman 2003). A U.S. National Toxicology Program evaluation panel concluded that acrylamide causes decreased birth weight in rodents (Manson et al. 2005), although the mechanisms underlying the effects of acrylamide on birth weight are not understood. Acrylamide is classified as a probable carcinogen to humans on the basis of animal studies and genotoxicity (IARC 1994), and its genotoxicity is thought to be attributable largely to metabolic conversion to the genotoxic epoxide glycidamide (Rice 2005). Acrylamide Hb adduct levels in adults were associated with decreased serum insulin and reduced insulin resistance (Lin et al. 2009). In addition, oxidative stress causing increased production of reactive oxygen radicals and inflammation was reported in 14 healthy volunteers after ingestion of 160 g of fried potato crisps daily for 4 weeks, which correspond to a daily acrylamide exposure that is approximately three times higher than the currently calculated ingestion of approximately 50 µg/day in the Western diet (Naruszewicz et al. 2009). The ability of acrylamide to readily react with sulfhydryl and amino residues in proteins, including enzymes, receptors, and cytoskeletal proteins, can affect a multitude of cellular processes and has been suggested to form the basis of some of acrylamide’s toxic effects (IARC 1994) and may contribute to the associations with birth outcomes observed in our study population.

The potential public health implications of our findings are substantial. Increases in head circumference are an important indication of continued brain growth, and reduced birth head circumference has been associated with delayed neurodevelopment (Gale et al. 2006). Reduced birth weight is a risk factor for numerous adverse health effects early in life, and has been associated with multiple adverse outcomes later in life, such as reduced stature, increased incidence of cardiovascular disease, type 2 diabetes mellitus, and osteoporosis (Gluckman et al. 2008). The estimated difference in mean birth weight among children in the highest acrylamide-exposed quartile compared with children in the lowest quartile was around 100 g, consistent with the reduction in birth weight observed for children exposed *in utero* to maternal smoking (Li et al. 1993). Many commonly consumed foods—fried potatoes and related products, crisp bread, and coffee—contain high concentrations of acrylamide (EFSA 2010; JECFA 2011; Manson et al. 2005; Tareke et al. 2002). The amount of acrylamide in specific foods varies widely depending on precursor levels and processing, and, crucially, is amenable to appropriate interventions in the preparation of food.

In summary, this large population-based study provides the first epidemiological evidence of a significant association between prenatal exposure to acrylamide and reduced birth weight and head circumference. If confirmed in other studies, these findings provide evidence supporting the need for changes in food production and for providing clear public health advice to pregnant women to reduce their dietary intake of foods that may contain high concentrations of acrylamide.

## Appendix: NewGeneris Consortium Collaborators

Victoria J. Burley,^1^ Ramon Carreras,^2^ Vincenzo Fontana,^3^ Theo M. de Kok,^4^ Margaretha Haugen,^5^ Kari Hemminki,^6^ Micheline Kirsch-Volders,^7^ Antonis Koutis,^8^ Martinus Løvik,^9^ Patricia A. McKinney,^10^ Helle M. Meltzer,^5^ Renee Mijal,^10^ Elena Stagi,^3^ Simone G. J. van Breda,^11^ Christopher P. Wild.^10^

## Correction

Some of the values in Table 4 were incorrect in the manuscript originally published online. They have been corrected here.

1School of Food Science and Nutrition, University of Leeds, Leeds, United Kingdom; 2IMIM (Hospital del Mar Research Institute), Barcelona, Spain; 3Epidemiology, Biostatistics, and Clinical Trials, National Cancer Research Institute, Genoa, Italy; 4Department of Toxicogenomics, Maastricht University, Maastricht, the Netherlands; 5Department of Food Safety and Nutrition, Division of Environmental Medicine, Norwegian Institute of Public Health, Oslo, Norway; 6Division of Molecular Genetic Epidemiology, German Cancer Research Center, Heidelberg, Germany; 7Laboratory of Cell Genetics, Faculty of Science and Bio-engineering, Vrije Universiteit Brussel, Brussels, Belgium; 8Department of Social Medicine, Faculty of Medicine, University of Crete, Heraklion, Greece; 9Department of Environmental Immunology, Norwegian Institute of Public Health, Oslo, Norway; 10Leeds Institute of Genetics, Health and Therapeutics, University of Leeds, Leeds, United Kingdom; 11National School of Public Health, Athens, Greece.

## Supplemental Material

(647 KB) PDF
