# Supplementary material for: Birth Weight, Head Circumference, and Prenatal Exposure to Acrylamide from Maternal Diet: The European Prospective Mother–Child Study (NewGeneris)
Source: Environ Health Perspect. 2012 Oct 23;120(12):1739–45. doi: 10.1289/ehp.1205327 (PMC3548277; doi:10.1289/ehp.1205327)
Supplement: (647 KB) PDF [file ehp.1205327.s001.pdf]

Supplementary Material

Birth Weight, Head Circumference, and Prenatal Exposure to Acrylamide from Maternal Diet: The European Prospective Mother-Child Study (NewGeneris)

Marie Pedersen, Hans von Stedingk, Maria Botsivali, Silvia Agramunt, Jan Alexander, Gunnar Brunborg, Leda Chatzi, Sarah Fleming, Eleni Fthenou, Berit Granum, Kristine B Gutzkow, Laura J Hardie, Lisbeth E Knudsen, Soterios A Kyrtopoulos, Michelle A Mendez, Domenico F Merlo, Jeanette K Nielsen, Per Rydberg, Dan Segerbäck, Jordi Sunyer, John Wright, Margareta Törnqvist, Jos C Kleinjans, Manolis Kogevinas and the NewGeneris Consortium

Table of Contents

Recruitment and ethical approval……………………………………………………………...1

Supplemental Materials, Table S1. Study protocol on recruitment and data collection…........2

Recruitment and ethical approval

Pregnant women were enrolled in Denmark, England, Greece, Norway and Spain, 2006-2010 (Supplementary Material, Table S1). The study was approved by the Regional Ethical Review Board in Stockholm, Sweden (Reference No. 2007/631-31).

The Danish biobank studies were approved by the Capital Region of Denmark (Reference No.: J. Nr. H-KF-01-327603; J. Nr. KF-11-260063 and J. Nr. H-B-2009-030) and notified to the Danish data protection agency (Reference No.: J. Nr. 2007-41-0415 and J. Nr. 2009-41-3763).

The RHEA studies were approved by the ethical committee of the University Hospital in Heraklion, Crete, Greece (Reference No.: 96-6/2/2007).

The MoBa sub-cohort BraMat studies were approved by the Norwegian Regional Committee for Medical and Health Research Ethics (Reference No.: S-06437a) and notified to the Data Inspectorate (Reference No.: 07/00154). The MoBa sub-cohort BraMiljø studies, has been approved by the Regional Committee for Medical Research Ethics (S-07133a) and the Data Inspectorate in Norway (07/00396-3/rvb).

The INMA sub-cohort studies were approved by the Clinical Research Ethics Committee (CEIC; Comité Ético de Investigación Clínica) of Barcelona (Reference No.: 2005/2106/1). At this time notification to the Spanish Data Protection Agency was not required.

The BiB sub-cohort studies were approved by the Bradford Local Research Ethics Committee, Bradford, the United Kingdom (Reference No.: 07/H1302/65) and also participants were part of the Born in Bradford main birth cohort.

Supplemental Material, Table S1. Study protocol on recruitment and data collection

Location	Period of recruitment	Methods of recruitment	Hospital(s), City	Inclusion criteria	Maternal blood	Questionnaires	Follow-up
Denmark, Copenhagen	December, 06 to December, 07	Written & oral invitation in end pregnancy	University Hospital of Copenhagen, Greater Copenhagen	>18 years<br>Singleton deliveries planned<br>Caesarean section deliveries<br>Wednesdays, 8 am to 12 am	Pre-delivery (day of delivery)	Full pregnancy<br>Collected around birth<br>Partly supported[1]	No
	September, 09 to December, 09	Oral & written invitation in end pregnancy	University Hospital of Copenhagen, Greater Copenhagen	>18 years<br>All deliveries<br>Monday to Friday	Pre-and post-delivery (day of delivery)	Full pregnancy<br>Collected after birth<br>Self administrated	Yes
Greece, Heraklion	February 07 to February 08	Oral invitation<br>at the first prenatal visit at or before week 15 of pregnancy	Venizeleion Hospital, University Hospital, Mitera maternity clinic, Asklipieion Hospital, Heraklion	> 16 years<br>All deliveries<br>Residence in study area<br>No communication handicap<br>All days	Pre- and post-delivery<br>(day of delivery)	First half of pregnancy<br>Completed at mid pregnancy<br>Administered by a trained research nurse	Yes
Norway, Oslo and Akershus	April, 07 to March, 08	Written invitation of women already enrolled in MoBa (The Norwegian Mother and Child study) in week 37 of pregnancy	Akershus and Ulleval University Hospital, Akerhus and Oslo	All ages<br>All deliveries<br>All days	0 to 3 days post-delivery	Full pregnancy<br>Collected in week 22 and 37<br>Self administrated	Yes
	October, 07 to July, 08		Ulleval University Hospital, Oslo	All ages<br>All deliveries<br>Monday to Friday<br>5 am to 6 pm	0 to 2 days post-delivery		No
Spain, Sabadell	May, 07 to June, 07	Oral invitation<br>in end pregnancy	Hospital Parc Taulí, Sabadell	>15 years<br>Singleton deliveries Monday to Friday,<br>7 am to 10 pm	Pre-delivery<br>(day of delivery)	Full pregnancy<br>Collected at the time of delivery<br>Partly supported	Yes
Barcelona	October, 08 to March, 10	Oral invitation<br>at the time of delivery	Hospital del Mar, Barcelona	>16 years<br>Singleton deliveries Monday to Fridays<br>4 am to 4 pm	Post-delivery<br>(day of delivery)		No
England, Bradford	January, 08 to December, 09	Written and oral invitation to the Born in Bradford study<br>in 26 to 28 weeks of pregnancy	Bradford Royal Infirmary, Bradford	>16 years<br>Planned Caesarean section deliveries<br>Monday to Friday	Post-delivery<br>(day of delivery)	Full pregnancy<br>Collected in weeks 26 to 28 and during last 4 weeks of pregnancy<br>Partly supported	Yes

[1] Partly supported by trained interviewers.
